# Army Medical Corps Examinations

**Published:** 1913-12

**Authors:** 


Army Medical Corps Examinations.—The Surgeon General of the Army announces that preliminary examinations for appointment of First Lieutenants in the Army Medical Corps will be held on January 19, 1914, at points to be hereafter designated.

Full information concerning these examinations can be procured upon application to the "Surgen General, U. S. Army, Washington, D. C." The essential requirements to secure an invitation are that the applicant shall be a citizen of the United States, shall be between 22 and 30 years of age, a graduate of a medical school legally authorized to confer the degree of Doctor of Medicine, shall be of good moral character and habits, and shall have had at least one year's hospital training as an interne, after graduation. The examinations will be held simultaneously throughout the country at points where boards can be convened. Due consideration will be given to localities from which applications are received, in order to lessen the traveling expenses of applicants as much as possible.

In order to perfect all necessary arrangements for the examinations, applications must be completed and in possession of The Adjutant General at least three weeks before the date of examination. Early attention is therefore enjoined upon all intending applicants. There are at present twenty-six vacancies in the Medical Corps of the Army.

